# Establishment and genomic characterization of gingivobuccal carcinoma cell lines with smokeless tobacco associated genetic alterations and oncogenic PIK3CA mutation

**DOI:** 10.1038/s41598-019-44143-0

**Published:** 2019-06-04

**Authors:** Kshama Pansare, Nilesh Gardi, Sayee Kamat, Prerana Dange, Rahul Previn, Poonam Gera, Pradnya Kowtal, Kishore Amin, Rajiv Sarin

**Affiliations:** 1 ICGC Lab, Advanced Centre for Treatment, Research and Education in Cancer, Tata Memorial Centre, Kharghar, Navi Mumbai, 410210 India; 2 Department of Medical Oncology, Tata Memorial Hospital, Tata Memorial Centre, Navi Mumbai, 410210 Maharashtra India; 3 Biorepository, Advanced Centre for Treatment, Research and Education in Cancer, Tata Memorial Centre, Kharghar, Navi Mumbai, 410210 India; 4 Sarin Lab, Advanced Centre for Treatment, Research and Education in Cancer, Tata Memorial Centre, Kharghar, Navi Mumbai, 410210 India; 5 Homi Bhabha National Institute, Training School Complex, Anushakti Nagar, Mumbai, 400094 Maharashtra India

**Keywords:** Cancer genomics, Cancer genomics, Oral cancer, Oral cancer

## Abstract

Smokeless tobacco associated Gingivobuccal squamous cell carcinoma (GB-SCC) is a major public health problem but available oral cancer cell lines are mostly from smoking associated tongue SCC raising the need for pertinent GB-SCC cell line models. As part of the International Cancer Genome Consortium (ICGC) Project, 4 novel cell lines, namely, Indian Tata Memorial Centre Oral Cancer (ITOC) −01 to −04 were established and characterized with conventional methods, karyotyping, ultrastructure, *in vivo* tumourigenicity, Whole exome sequencing (WES) and RNA sequencing. These hyperploid cell lines form xenografts in mice and show metabolically active and necrotic areas on fluorodeoxyglucose-positron emission tomography (FDG-PET) imaging. WES of ITOC cell lines recapitulate the genomic tumor profile of ICGC GB-SCC database. We further identified smokeless tobacco associated genetic alterations (*PCLO, FAT3* and *SYNE2*) and oncogenic *PIK3CA* mutation in GB-SCC cell lines. Transcriptome profiling identified deregulation of pathways commonly altered in cancer and down-regulation of arachidonic acid metabolism pathway, implying its possible role in GB-SCC. Clinical application of high throughput sequencing data depends on relevant cell line models to validate potential targets. Extensively characterized, these oral SCC cell lines are particularly suited for mechanistic studies and pre-clinical drug development for smokeless tobacco associated oral cancer.

## Introduction

Advanced stage oral cancer with poor clinical outcome is a major public health problem in south Asian and east Asian countries where smokeless tobacco use is prevalent^1,2^. Smokeless tobacco is consumed in various forms with areca nut, lime or betel leaves as quid, pan, masheri or gutka^3,4^. Most oral cancers are squamous cell carcinomas (SCC) and arise from the lip, tongue, gingivobuccal mucosa, retromolar trigone, floor of mouth or the hard palate. The form of tobacco use not only influences the site of cancer within the oral cavity but also its biology and mutational profile. In western countries where smoking is the predominant form of tobacco use, tongue cancer is the most common oral cancer while in south Asian and east Asian countries where tobacco chewing is more prevalent, Gingivobuccal SCC (GB-SCC) is the most common oral cancer. GB-SCC differs from the tongue SCC in its clinical behaviour^5^ as well as molecular^6–8^ and immunoproteome profile^9^, yet the treatment modalities are identical. Previous studies have revealed that as compared to tobacco smoking, the smokeless tobacco associated cancers have predominance of specific transversions or transitions in *TP53* and the genome^10–12^.

Comprehensive molecular genetic characterization using whole genome, exome and transcriptome analysis has been reported in over 400 Head and Neck SCC (HNSCC) tumours. These include GB-SCC by our group as part of the International Cancer Genome Consortium (ICGC) India Project^6^ and The Cancer Genome Atlas (TCGA) project in USA for diverse HNSCC^13^. Large number of novel and recurrently mutated genes and deregulated pathways in HNSCC, with some being unique to GB-SCC were identified. Mechanistic studies on the role of an increasing number of genetic variants being identified in different cancers and their translational exploitation requires cell lines from same organ and histology with full molecular genetic characterization.

Primary cultures and immortalized cell lines are indispensible source for conducting translational cancer research. Tumor-derived cell lines are widely used *in vitro* models due to the ease of performing large scale experiments, genetic manipulation and reproducibility^14^. Cell lines maintained using appropriate conditions retain parent tumor features and phenotype, making them preferred tumor representative models in laboratory studies^15,16^. The dearth of commercial cell lines for tumours which are uncommon in large parts of the world necessitates the need to establish and characterize novel cell lines from primary tumor^17^.

Of the several hundred HNSCC cell lines reported worldwide, very few are from GB-SCC in patients with smokeless tobacco use^18–20^ and none from the Indian subcontinent. As part of our systematic efforts to establish relevant cell lines in the ICGC India oral cancer project, we have established and performed comprehensive molecular genetic characterization of 4 oral squamous cell carcinoma (OSCC) cell lines from tumour tissues obtained from tobacco chewing Indian patients. These cell lines are named ITOC-01, 02, 03 and 04, after the ethnic origin, institute and cancer site - **I**ndian **T**ata Memorial Centre **O**ral **C**ancer (ITOC). These cell line models established from smokeless tobacco consuming patients provide a platform to carry out wide spectrum of assays spanning from functional genomics to drug response studies. To the best of our knowledge this is the first study from Indian subcontinent characterizing smokeless tobacco GB-SCC derived cell lines.

## Results

### Cell line establishment and characterization

Details of the patients from whom these cell lines were derived are provided in Table 1. All the 4 cell lines are stably self immortalized and have under gone at least 70 passages. They have been maintained for over 4 years and cryopreserved at regular intervals.

**Table 1:** Patient’s demographic, tumour and treatment characteristics.

	ITOC-01	ITOC-02	ITOC-03	ITOC-04
Patient age, Gender & Ethnicity	28 years, Male, Indian	43 years, Female, Indian	37 years, Male, Indian	61 years, Male, Indian
Primary site	Buccal mucosa	Oral tongue	Buccal mucosa	Buccal mucosa
Histology (grade)	SCC (poorly differentiated)	SCC (poorly differentiated)	SCC (moderately differentiated)	SCC (poorly differentiated)
Pathological staging	T4N2cM0	T4aN0	T3N2bM0	T4aN2bM0
Habits	Chronic tobacco chewer using Gutka, Masheri, Paan with tobacco & Snuff. No smoking or alcohol.	Chronic tobacco chewer using Masheri. No smoking or alcohol.	Chronic tobacco chewer. No smoking or alcohol.	Chronic tobacco chewer using Gutka. No smoking or alcohol.
Treatment details	Radical surgery followed by radiotherapy (RT) + chemotherapy (CT)	Radical surgery followed by RT + CT	Radical surgery followed by RT + CT	Neoadjuvant Paclitaxel and Carboplatin chemotherapy followed by radical surgery. Subsequent adjuvant RT + CT
Treatment outcome	Rapid progression and died after 6 months	Rapid progression after 6 months of treatment	Disease controlled at 1 year follow up	Disease controlled at 6 month follow up

Masheri: Burnt tobacco applied over gums; Paan: Betel leaves with lime, catechu and areca nut.

### Morphology and ultrastructure analysis

**Figure 1:**
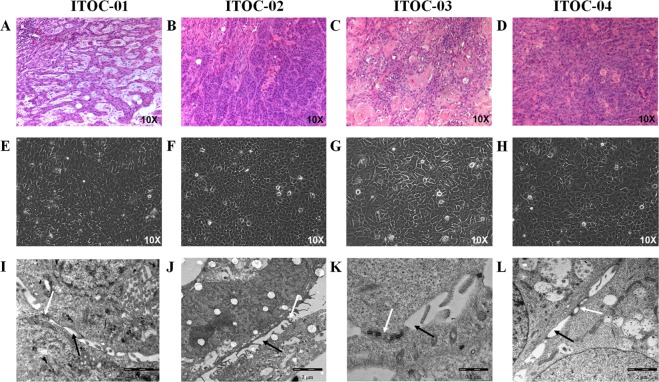
Morphology of OSCC cells in culture. (**A**–**D**) Histological sections of the primary oral tumors showing squamous cell carcinoma. (**E**–**H**) Phase contrast photomicrographs of cells in culture at 10X magnification. (**I**–**L**) TEM images of all the four cell lines exhibiting desmosomes (white arrow) and intracellular spaces (black arrow).

**Figure 2:**
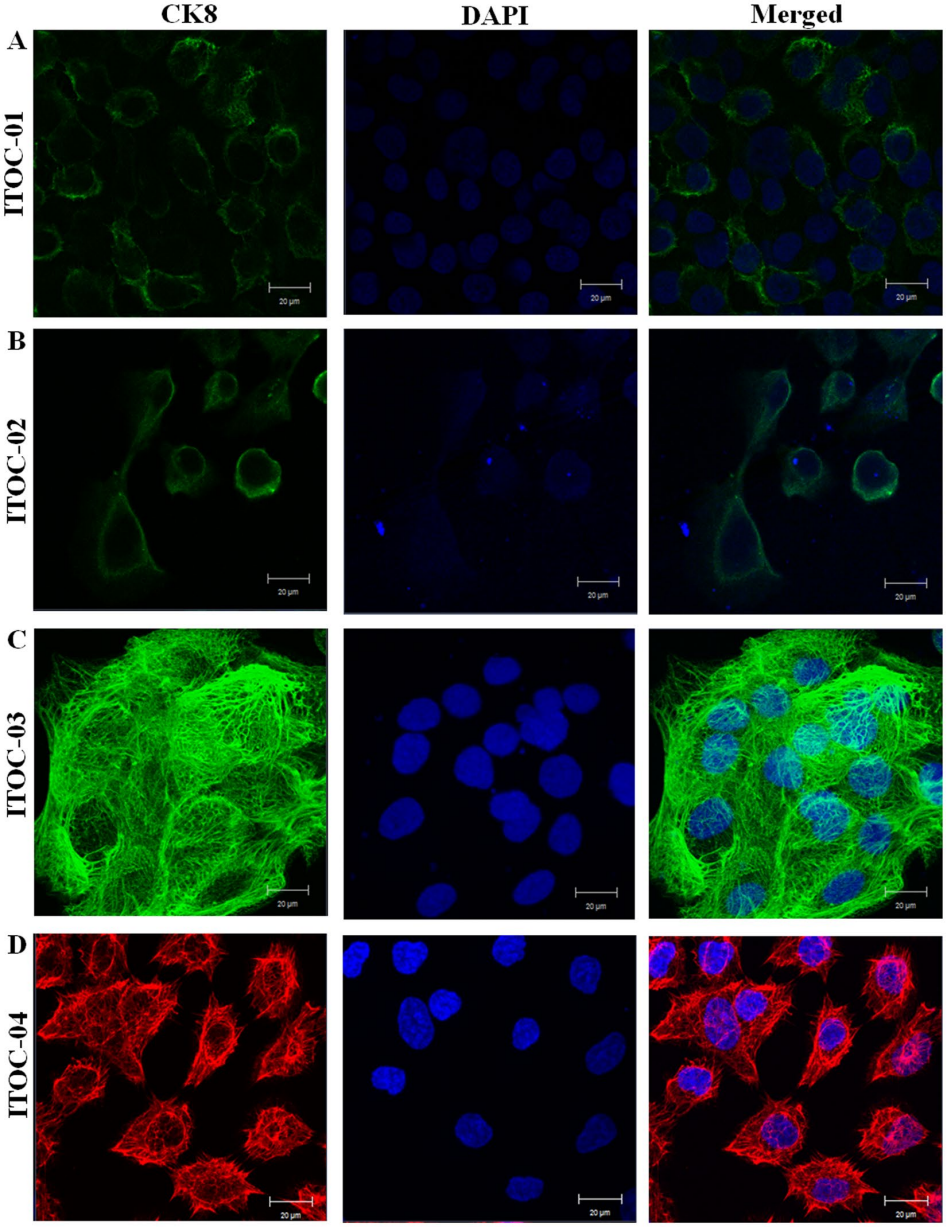
(**A**–**D**) Confocal micrographs showing the expression of epithelial membrane antigen CK8 in OSCC derived cell lines. ITOC-01, ITOC-02, ITOC-03 cell lines were probed with anti-mouse IgG-FITC secondary antibody, while Alexa Fluor 568 anti-mouse secondary antibody was used for ITOC-04 cell line.

Classical epithelial morphology and characteristic SCC features were seen in all four parent tumors (Fig. 1A–D). Detailed histological evaluation of OSCC tumors is tabulated in Supplementary Table S1. ITOC cells were mononuclear and appeared flat and polygonal in shape. ITOC-01 cells adhered to each other forming clumps while ITOC-02, ITOC-03 and ITOC-04 cells grew in a monolayer (Fig. 1E–H). ITOC cells morphology is indicative of squamous epithelial cells, hence suggesting the squamous origin of the parent tumor being retained in all the 4 OSCC cell lines. Transmission electron microscopy (TEM) revealed studded desmosomes on the adjacent cell membranes, characteristic of epithelial morphology, condensed nuclear chromatin, irregular nuclear envelope and other cytoplasmic organelles (Fig. 1I–L). Bundles of intermediate filament in cytoplasm and microvilli on the cell surface were seen. Membranous staining for epithelial marker cytokeratin 8 confirmed epithelial origin of all 4 cell lines (Fig. 2A–D).

### DNA profiles

Genotyping confirmed the human origin and unique genotype of each cell line (Supplementary Table S2). Comparison with Children’s Oncology Group (COG) and American Type Tissue Culture (ATCC) short tandem repeat (STR) databases showed no significant relatedness with any of the established cell lines affirming unique origin of each cell line. Apparent gender mismatch was observed in ITOC-04 cell line established from a male patient. The Y allele was not detected in the Amelogenin marker in the cell line but both X and Y alleles were intact in the normal and tumor tissue of this patient. Loss of Y allele during cell passaging is a known phenomenon^21^.

### Chromosome analyses

**Figure 3:**
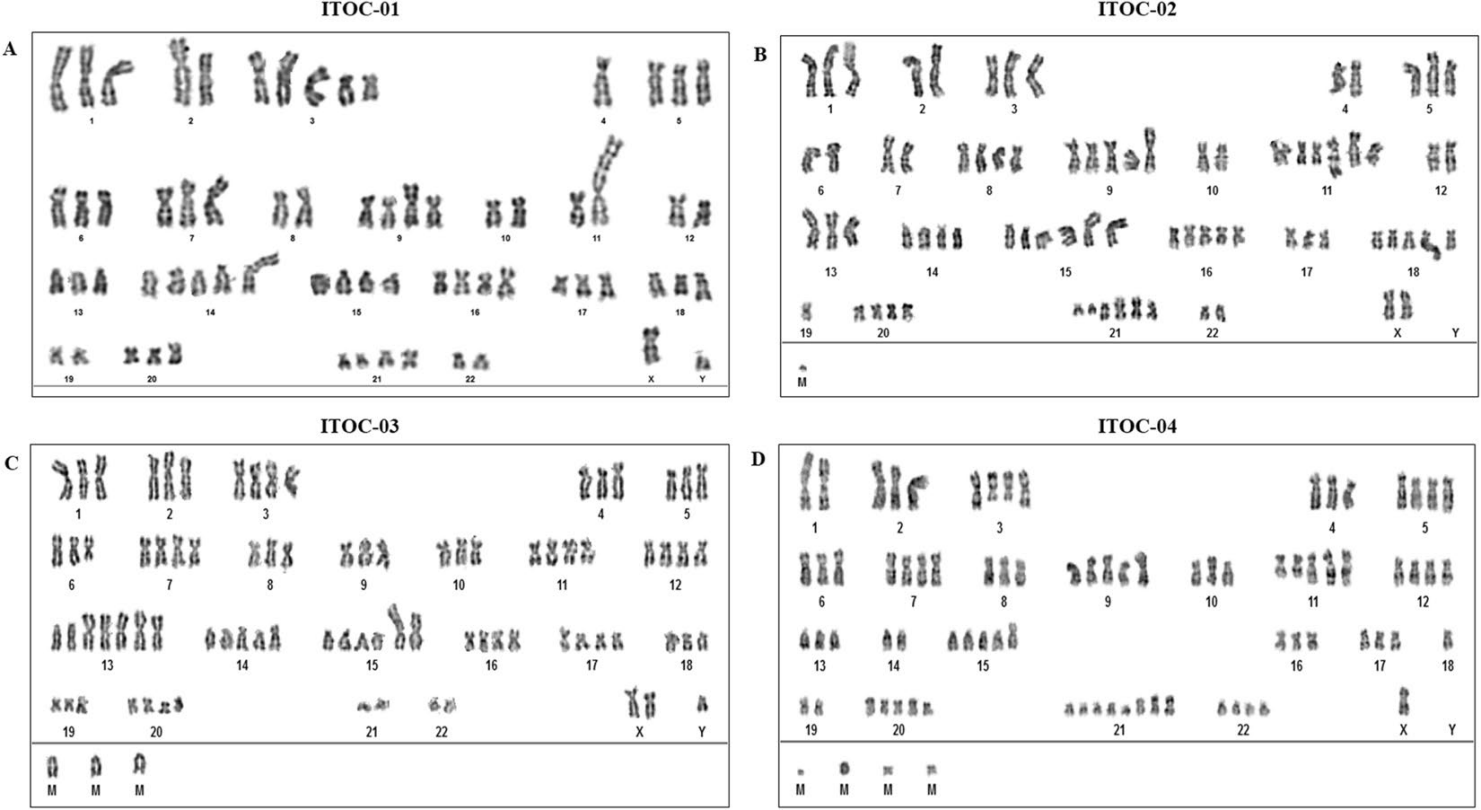
Chromosomal analysis of OSCC cell lines. (**A**) Composite karyotype of ITOC-01 cell line: 63 ∼ 70,XY, +der(1)(p36), +der(2)t(2;6)(p24;q13), +der(3)del(3)(p10)x2,4, +5, +6,dup(7)(q11.2q22), +der(7)t(4;7)(q21;p22),del(7)(q32), +9, +der(9)t(9;?13)(p23;?q10), +10, +10, +der(11)t(4;11)(pterq26;p15),add(12)(p13), +13, +14, +14,der(14)t(13;14)(q10;q10), +15, +der(15)add(15)(p11.5), +16, +17,der(18)dup(18)(q21.1;q23), +20, +20,der(21)t(21;?)(q21;?),add(21)(q21)[cp20]. (**B**) Composite karyotype of ITOC-02 cell line: 72 ∼ 82,XX, +1, +3, +5, +8, +8, +9, +9,i(9)(q10;q10), +11, +11, +der(11)dup(11)(q13q23), +der(11)?t(11;?)(q;?),der(13)t(13;14)(q10;q10)x2, +der(13)t(13;?)(q10;?), +14, +14, +15, +der(15)t(14;15)(q10;q10)x3, +16, +16, +16, +17, +18, +18, +der(18)t(11;18)(q23;q23),19, +20, +20, +, +der(21)t(17;21)(q21;q22),der(21)t(21;21)(q10;q22)x3, +mar[cp20]. (**C**) Composite karyotype of ITOC-03 cell line: 80 ∼ 89,XY, +X, +1, +2, +3, +3, +4, +5, +der(6)t(6;?)(q10;?), +7, +7, +8, +9, +10, +11, +11, +12, +12, +der(13),t(13;14)(q10;q10)x2, +der(13)t(13;13)(q10;q10)x3, +14, +, +14, +der(14)add(14)(q11.2), +15, +15, +der(15)t(15;?)(q11.2;?)x2, +16, +16, +17, +17, +18, +19, +20, +20, +mar x3[cp20]. (**D**) Composite karyotype of ITOC-04 cell line: 84 ∼ 93,X,der(1)dup(1)(q22p33); +der(2)add(2)(p22p23), +der(3)t(3;?)(p21;?)x2, +4, +5, +5, +6, +7, +7, +8, +9, +9,i(9)(q10;q10), +der(10)t(10;?)(p10;?),der(11)dup(11)(q23)x3, +12, +12, +13, +15, +15,der(15)dup(15)(p10), +16, +17,18, +20, +20, +20, +21, +21, +21, +21, +der(21)t(21;21)(q10;q10), +22, +22, +mar1, +mar2x2, +mar3[cp20].

Chromosomal analyses of ITOC-01, ITOC-02, ITOC-03 and ITOC-04 cell lines showed complex near triploid, hypertriploid, hypotetraploid, and near tetraploid karyotype respectively. Human male karyotype was observed in ITOC-01 and -03 cell line whilst ITOC-02 cell line showed human female karyotype. Loss of Y allele was observed in ITOC-04 cell line as seen in STR marker analysis. Aneuploidy was observed in all cell lines wherein the chromosome number ranged between 63–93 chromosomes (Fig. 3A–D).

### Growth characteristics and DNA content

Analysis of cell proliferation was carried out using growth curve. Population doubling time of ITOC-01, 02, 03 and 04 cell line was 47.31, 51.15, 67.82 and 47.75 hours respectively (Supplementary Fig. S1A–D). DNA histogram analysis of ITOC-01, ITOC-02, ITOC-03 revealed hyperploidy with DNA indices of 1.6, 1.8, 1.2, respectively, while ITOC-04 revealed tetraploidy, with DNA index of 2.0 (Supplementary Fig. S1E–H).

### Transfection efficiency

Transfection efficiency of OSCC cell lines was evaluated by transfecting enhanced green fluorescent protein expression plasmid (pEGFP-C1) with GeneJuice transfection reagent. EGFP expression observed after 48 hours of transfection revealed low transfection efficiency in ITOC-01 (1%) and ITOC-02 (6%) but high efficiency in ITOC-03 (30%) and ITOC-04 cell lines (20%) (Supplementary Fig. S2).

### Scratch assay and anchorage independent growth

At 24 hours, cell migration calculated using ImageJ software showed 24%, 37%, 51% and 100% wound healing for ITOC-01, ITOC-02, ITOC-03 and ITOC-04 cell lines respectively. One-way ANOVA (analysis of variance) and Bonferroni post-tests showed high significance between control and time points of 24 and 48 hours (Supplementary Fig. S3A). At 21 days, anchorage independent growth in ITOC-01 and ITOC-02 showed large colonies while ITOC-03 and ITOC-04 showed small colonies (Supplementary Fig. S3 B–E).

### *In vivo* tumourigenicity and fluorodeoxyglucose - positron emission tomography (FDG-PET) imaging

**Figure 4:**
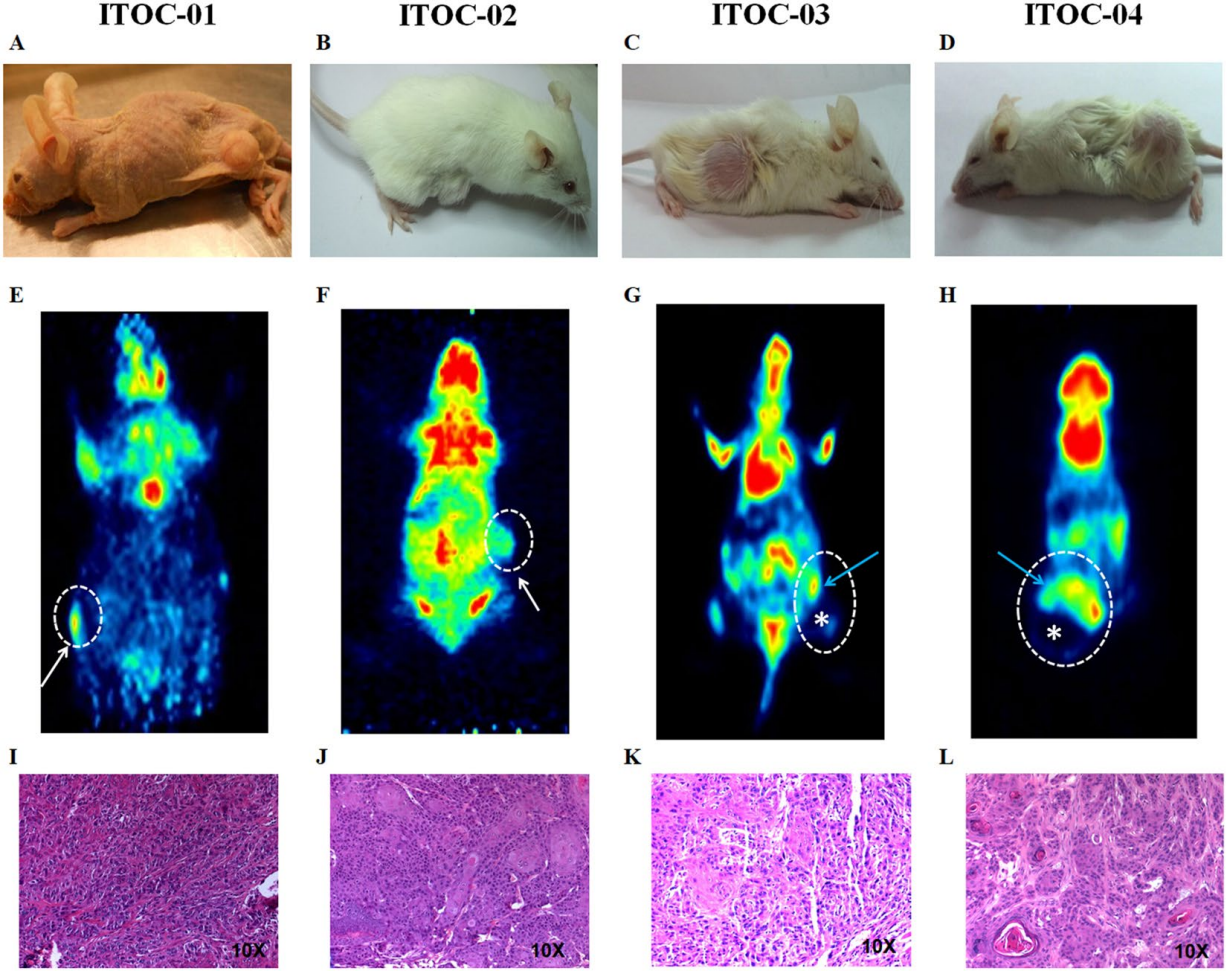
Tumourigenicity of OSCC cell lines. (**A**–**D**) Subcutaneous tumor formation in nude (ITOC-01) and NOD SCID mice (ITOC-02, ITOC-03, ITOC-04) after inoculation of OSCC cell lines. (**E**–**H**) Micro PET images of 18-FDG uptake in coronal planes for cell line xenografted mice. Tumor is indicated with white broken line with areas of necrosis depicted with *. Blue arrow indicates the viable base tumor in PET scan. (**I**–**L**) (**H**&**E**) stained microphotographs from tumor xenografts captured at 10X magnification.

ITOC-01 cell line developed subcutaneous tumor in the dorsal flank region in NUDE mice in 6 weeks, while ITOC-02, ITOC-03 and ITOC-04 cell line developed subcutaneous tumor in nonobese diabetic – severe combined immunodeficiency (NOD-SCID) mice in 3, 2 and 2 weeks respectively (Fig. 4A–D). Grossly, the xenograft tumors were firm, grey-white and partially necrotic with whitish thick exudate. Histological analysis was done on the sections taken from grey-white firm areas revealing characteristic squamous cell carcinoma features. Distant metastasis was not observed on FDG-PET scan (Fig. 4E–H) or on histology done after subcutaneous injection in any of the animals (Fig. 4I–L, Supplementary Table S1). Histopathological evaluation of primary and respective xenograft tumors revealed squamous cell carcinoma of varying grades. Detailed morphological analysis of the tumors confirmed characteristic features of the parent tumor being retained in the xenograft tumors (Supplementary Table S1).

### Exome analysis

**Figure 5:**
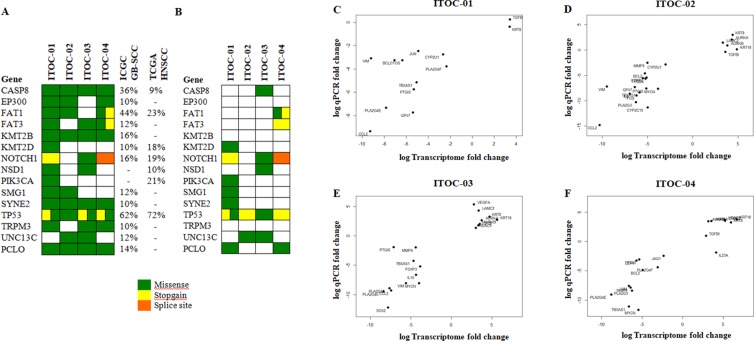
Mutational profile and validation of RNA-seq data. (**A**) Somatic nonsynonymous variants in genes recurrently mutated in ≥10% ICGC GB-SCC or TCGA HNSCC samples. (**B**) Somatic truncating and splice site mutations and missense variants predicted to be deleterious by ≥5 functional prediction tools. *Variants classified as deleterious by 4 prediction tools. (**C**–**E**) Validation of RNA-seq data by qPCR. 30 genes identified to be differentially expressed across OSCC cell lines were validated by Real time qRT-PCR. Comparison of gene expression values of RNA-seq and qPCR showed concordance in the data.

Whole exome sequencing (WES) was done at a mean depth of 155x, 112x, 128x and 113x for ITOC-01, -02, -03 and -04 cell line respectively with mean mapping quality of 58.12. A total of 24150, 21921, 22576 and 22018 exonic variants were identified in the ITOC-01, 02, 03 and 04 cell lines respectively, of which, 369, 265, 343 and 312 somatic variants were considered deleterious. Each cell line had a mutational profile matching with the GB-SCC or HNSCC mutational profile reported in the ICGC GB-SCC and TCGA HNSCC database (Supplementary Table S3, S4). Of the 15 most recurrently mutated genes in ≥10% of HNSCC or GB-SCC tumours as reported in the TCGA and ICGC database (*TP53, FAT1, CASP8, NOTCH1, KMT2B, PCLO, UNC13C, SMG1, FAT3, EP300, KMT2D, SYNE2, TRPM3, PIK3CA* and *NSD1*), 12 were mutated in one or more of the ITOC cell lines (Fig. 5A,B and Supplementary Table S5). All 4 cell lines had *TP53* mutation while 3 cell lines had *NOTCH1* deleterious mutations. The somatic variants mainly belonged to the apoptosis (*TP53, BMP3, BMP4, TRAF5, AVEN*) and inflammatory pathways (*IKBKB, MMP10, CYP2A6, CYP4B1*), implying their role in cancer progression^22–29^. Mutational signature in the 4 cell lines were similar with C > T transitions (44%) being most common, followed by T > C (20%), C > A (21%) and C > G (15%) transversions (Supplementary Fig. S4).

Comparative genomic analyses of parent tumor, cell lines at early passage (ITOC-01: P-10, ITOC-02: P-8, ITOC-03: P- 12, ITOC-04: P-9), cell lines at late passage (ITOC-01: P-51, ITOC-02: P-48, ITOC-03: P-58, ITOC-04: P-57) and xenograft tumors was performed by evaluating *TP53* mutational status, the most frequently mutated gene in HNSCC and which is also found to be mutated in all 4 OSCC cell lines (Supplementary Fig. S5). We identified R196X nonsense mutation and Y126H missense mutation in ITOC-01, R306X nonsense mutation in ITOC-02, IVS2 + 38 C > G mutation in ITOC-03 and ITOC-04 and IVS3 + 36_52del16 and R342X in ITOC-04 parent tumor, cell line at early and late passage and xenograft tumors. However, in ITOC-02, the R306X mutation was seen in the cell line and primary tumour but not in the xenograft, possibly due to the heterogeneity in the xenograft.

### Transcriptome analyses

RNA-seq of three normal buccal mucosa tissues and four OSCC cell lines generated ~65 million reads each. Compared to the 3 normal buccal mucosa tissues, 661, 811, 885, 907 genes were upregulated and 1627, 1997, 2343, 1950 genes down-regulated in ITOC-01, -02, -03 and -04 cell lines respectively (Supplementary Tables S6, S7). Gene Set Enrichment Analysis (GSEA) with Kyoto Encyclopedia of Genes and Genomes (KEGG) gene sets identified Notch signaling, p53 signaling, arachidonic acid metabolism (AAM), mitogen activated protein kinase (MAPK), chemokine, focal adhesion, extracellular matrix (ECM) receptor interaction, cell cycle, DNA replication pathway as significantly deregulated pathways in one or more of these 4 OSCC cell lines (Supplementary Fig. S6). Analysis limited to the 3 GB-SCC cell lines (ITOC-01, 03 and 04) identified similar deregulated pathways (Supplementary Fig. S7).

RNA-seq data was validated by quantitative polymerase chain reaction (qPCR) for 30 genes which are known to be deregulated in cancer and also in these cell lines (Fig. 5C). Gene expression of individual cell lines was normalized to normal buccal mucosa tissue using *β-actin* endogenous control. Up-regulation of keratins (*KRT8,18*) and down-regulation of Vimentin validates the epithelial origin of these cell line and also corroborates with the earlier result of expression of keratin 8 in OSCC cell lines (Fig. 2A–D). The AAM pathway genes which are mutated in GB-SCC (*PLA2G3, PLA2G4E, PLA2G4F, PTGIS, TBXAS1, CYP2U1, CYP2C19, GPX7*) were down-regulated in ITOC cell lines.

## Discussion

Human cancer cell lines are indispensable to functionally characterize new or previously uncharacterized genetic alterations identified by large scale genomic studies. In the precision medicine era, the utility of a cell line for identifying clinically relevant targets requires these cell lines to closely recapitulate the molecular genetic profile of specific human cancers. Despite the common SCC histology, prior tobacco exposure and several common genetic alterations, the carcinomas arising at different primary sites within the Head and Neck region have several distinct features^7,8,18^. The Expert protein analysis system (ExPASy) – Cellosaurus database has reported over 150 SCC cell lines derived from tongue cancer but very few from buccal mucosa^30–32^ or gingiva^33–35^. Surprisingly, no GB-SCC cell lines are reported in literature or databases from the Indian subcontinent where it is the most common oral cancer. One possible reason may be the difficulty in culturing and establishing cell lines from heavily infected locally advanced gingivobuccal cancers.

We have successfully established and characterized 4 OSCC cell lines including 3 from GB-SCC derived from tobacco chewers. We demonstrate these cell lines to be of human origin with characteristic features of SCC. Identification of identical *TP53* mutations across parent tumor, cell line at early and late passage and xenograft tumors affirms the genomic characteristics of primary tumor being preserved throughout the cell line cultivation and further in the xenograft model as well. The genomic and histopathological analyses assure the OSCC cell lines are representative models of the parent tumors. Through exome and transcriptome analysis we confirm that ITOC cell lines recapitulate the mutational and gene expression profile of GB-SCC and HNSCC in the ICGC and TCGA studies^6,13^. Of the 15 most recurrently mutated HNSCC or GB-SCC genes, 8 genes (*PCLO, SYNE2, FAT3, EP300, KMT2B, SMG1, TRPM3 and UNC13C*) were mutated in >10% of smokeless tobacco associated GB-SCC tumours of ICGC^6^ but not in the cigarette smoking associated HNSCC of TCGA study^13^ and may be considered as smokeless tobacco associated genes (Fig. 5A,B). Three of these 8 smokeless tobacco associated genes (*PCLO, SYNE2 and FAT3*) had deleterious mutation in GB-SCC ITOC cell lines (ITOC-01, 04) but not in the Tongue-SCC ITOC-02 cell line. This highlights the need for establishing, characterizing and using cell lines from tumours of the same subsite within HNSCC that have developed after similar form of tobacco exposure. Interestingly, the tongue SCC ITOC-02 cell line had truncating mutations in a few genes which are rarely associated with some cancers, *PYROXD2, PCMTD1* reported to be mutated in hepatocellular cancer^36,37^ and *PABCP1* reported to be upregulated in gastric carcinoma and targeting miR-34c^38^.

Moreover, ITOC-01 is the only GB-SCC cell line harbouring an oncogenic *PIK3CA* mutation (H1047R). It would be particularly useful to study *PIK3CA* inhibitors like Alpelisib (BYL719) which has been evaluated in only 2 *PIK3CA* mutated HNSCC cell lines^39^. Encouraging results from first in human use of Alpelesib in diverse tumours with oncogenic *PIK3CA* mutations has been reported^40^ and merits further evaluation in HNSCC including GB-SCC.

Earlier studies have proposed genetic signature 29 with abundance of C > A transversion for smokeless tobacco associated cancers^10^, however, this was not observed in any of the 4 ITOC cell lines. Similar to our finding, Fadlullah *et al*.^20^ had also not observed any correlation between the patient habits and mutation signatures. It is important to note that smokeless tobacco is used in many forms with or without lime, areca nut and other potentially carcinogenic ingredients. Hence there is a need to re-examine signature 29 and its association with smokeless tobacco and forms of smokeless tobacco use.

A common limitation of genetic profiling studies on cell lines is the absence of paired normal germline and gene expression data^20,41^. For the ITOC cell lines we used stringent bioinformatic methods to deduce somatic mutations. This seems to be a valid approach since the known mutational profile of HNSCC and GB-SCC is recapitulated in the ITOC cell lines. Since gene expression is tissue dependent, to interpret deregulated pathways in specific cancers, it is important to use representative normal tissues. In the TCGA HNSCC study with 16 GB-SCC tumours, their head neck region normal tissue dataset did not have any normal buccal mucosa sample for gene expression analysis. Similarly, the oral cancer cell line panel study used cultured keratinocytes in lieu of normal oral mucosa tissue^20^. Pure normal cell population in culture may be used as a convenient surrogate for normal tissues. However it is well known that isolation of specific cell populations, immortalization of normal or tumour cells, and the culturing conditions could all result in major alterations in gene expression as compared to their counterpart normal tissue or primary tumor^42^. This may result in misidentification of altered genes and pathways leading to false discovery. Possibly as a result of using normal buccal mucosa for determining differential gene expression, the deregulated genes in ITOC cell lines show excellent correlation with the qPCR data (Fig. 5C).

The ITOC cell lines show up-regulation of inducers of epithelial to mesenchymal transition (*VEGFA*)^43^, epigenetic markers (*AURKA, AURKB, HDAC9*); ECM receptor interaction molecules (laminin - *LAMC2*) and *IL23A*. A recent study has shown up-regulation of *LAMC2* as a marker of cancer invasion in oral leukoplakia^44^. We propose a similar role could be observed in OSCC-GB. ITOC cell lines also show up-regulation of ECM receptor interaction and focal adhesion pathway, similar to previous large scale transcriptomic analysis of 326 oral cancer tissues^45^. Previous report by ICGC India team has shown loss-of-function somatic mutations in AAM pathway leading to better patient survival^46^. Considering the identification of somatic mutations and down-regulation of gene expression in AAM pathway components of these novel cell lines it would be interesting to further elucidate the role of AAM pathway in OSCC.

This is the first report of comprehensive molecular genetic characterization of 4 OSCC cell lines established from Indian patients. The ITOC cell lines recapitulate the molecular genetic profile of smokeless tobacco associated GB-SCC and tongue SCC as identified by the ICGC and TCGA studies. Moreover, ITOC-01 is the only GB-SCC cell line with oncogenic *PIK3CA* mutation. Tumorigenic potential along with the genomic characterization make the ITOC cell lines particularly useful for mechanistic and translational studies and screening novel agents in a disease with high recurrence rates despite best multimodality treatment.

## Methods

### Tumor specimen collection and establishment of cell lines

This study was approved by Tata Memorial Centre (TMC) – Advanced Centre for Treatment, Research & Education in Cancer (ACTREC) Institutional Ethics Committee III (Project No. 3). This study was conducted at TMC, ACTREC and all experiments involving human tissue were performed in accordance with relevant guidelines and regulations. Tumor tissues were collected at the time of surgery after obtaining informed consent from patients with smokeless tobacco associated oral SCC. Tumor tissues were transported in 5 mL Plain Iscove’s Modified Dulbecco’s Medium (IMDM) (Gibco) with double strength antibiotic mixture of Penicillin 400 U/mL, Streptomycin 40 µg/mL, Gentamycin 50 µg/mL and Mycostatin 10 U/mL (PSGM). Tissues were processed within half an hour of resection and were rinsed twice with sterile 1X phosphate buffered saline (PBS) followed by 10% Povidone-iodine (Wockhardt) and PBS wash. Tumor tissues were minced finely to small pieces of approximately 1–2 mm^3^ in size and incubated for 48 hours in 4 mL of complete IMDM [90% plain IMDM and 10% fetal bovine serum (FBS) (Gibco) supplemented with antibiotic] at 37 °C in a humidified atmosphere containing 5% CO_2_. Culture medium was replaced every 48 hours and the epithelial pool of cells was enriched by differential trypsinization and subsequent removal of fibroblasts. Cells at 80% confluency were split in the ratio of 1:2 twice a week. Four cell lines were established – 3 from GB-SCC (ITOC-01, 03 and 04) and 1 from tongue SCC (ITOC-02).

### Phase contrast and electron microscopy

Morphological characteristics of the cell lines were studied using Olympus IX51 phase contrast microscope and ultra structure analysis was performed with Transmission Electron Microscope (JEM 1010, JEOL, Tokyo, Japan) at 120 kV (Supplementary Materials & Methods).

### Chromosome preparation and giemsa banding

Cells were harvested and arrested with colcemide (0.05 µg/mL) for 1 hour at 37 °C. Mitotic cells were centrifuged at 2000 rpm for 5 minutes followed by hypotonic KCl treatment for 30 minutes at 37 °C. Cells were fixed in fresh methanol: glacial acetic acid (3:1, v/v) for 15 minutes at 4 °C. G-banding was performed by treating the slides with trypsin followed by Giemsa staining (GTG). Metaphases were captured using Olympus BX61 microscope (Olympus, USA) and analyzed using Applied Spectral Imaging (ASI) GenASIs software version 7.0 (ASI, Israel).

### DNA profiling

Genomic DNA of cell lines was used for STR analysis of 8 autosomal markers (CSF1PO, D5S818, D7S820, D13S317, D16S539, THO1, TPOX, vWA) and 1 gender marker, Amelogenin. PCR products were analyzed with 3500 Genetic Analyzer (Applied Biosystems) and compared with COG STR database (http://strdb.cogcell.org) and ATCC STR database (http://atcc.org/STR_Database) of cell lines using match threshold of ≥80%.

### Cell proliferation assay and doubling time

Cells were seeded at a density of 5 × 10^3^ cells/well in 150 μL medium. 3-(4,5-dimethylthiazolyl-2)-2,5-diphenyltetrazolium bromide (MTT) solution was added at a final concentration of 0.5 mg/mL and incubated for 4 hours at 37 °C, 5% CO_2_. Purple formazan crystals were dissolved by addition of 10% SDS. Absorbance was measured at 540 nm with a spectrophotometer (BMG Labtech). Cell proliferation was assayed at different time intervals - 0, 24, 48, 72 and 96 hours. MTT readings were recorded in triplicates and the overall mean plotted against time. Standard error of the mean (SEM) were calculated for each day and the exponential growth curve plotted. Doubling time calculated using the equation ln(2)/K, where K is the growth rate constant.

### DNA measurement

Lymphocytes isolated from fresh blood of a healthy donor were used as control for ploidy studies. Cells fixed in 70% ethanol for 60 minutes were incubated with 100 µg/mL RNase A (Sigma-Aldrich) and stained with 50 µL propidium iodide (Sigma-Aldrich) for 30 minutes at 37 °C. Acquisitions were performed on BD FACSCalibur (Becton Dickinson) and data analyzed using Modfit software (version 2.0).

### Immunofluorescence staining

Cells were grown on sterile coverslips for 48 hours, fixed in ice-cold methanol at −20 °C for 10 minutes. Coverslips were rinsed with PBS and permeabilized using 0.3% Triton X and ice-cold methanol at room temperature (RT). Blocking was carried out using 3% bovine serum albumin (BSA) in PBS for 15 minutes at RT. Cells were incubated with primary antibodies for 1 hour at RT; p53 (Mouse monoclonal ab26, Abcam: 1:50), Cytokeratin 8 (Mouse monoclonal, Abcam, 1:100) followed by 1 hour incubation with fluorescein isothiocyanate (FITC) secondary antibody (Anti-Mouse IgG-FITC, Sigma), Alexa Fluor 568 goat anti-mouse IgG (H + L), Molecular Probes). Coverslips were mounted using Vectashield (Vector Labs) mounting medium with 4′,6-diamidino-2-phenylindole (DAPI) and viewed under confocal microscope (Carl Zeiss, LSM780).

### Transfection efficiency assay

Cells seeded at a density of 3 × 10^5^ in 35 mm cell culture dish were transfected with 1 µg of pEGFP-C1 plasmid using GeneJuice (Merck) transfection reagent as recommended by the manufacturer’s instructions. Cells were incubated at 37 °C for 48 hours and transfection efficiency was evaluated by counting 200 cells from five different fields under a fluorescent microscope.

### Cell migration assay

Cells were seeded at a density of 2 × 10^6^ in 6-well plate and on reaching 100% confluency a wound was inflicted with a pipette tip and images were captured every 24 hours on Olympus IX51 microscope and analyzed using ImageJ software. The relative cell free area at respective time points was calculated by considering the inflicted wound at 0 hours as 100%. One-way analysis of variance (ANOVA) and Bonferroni post-tests were performed to analyze effect of wound healing with respect to time.

### Anchorage independent growth

1 × 10^5^ cells/mL were suspended in 0.2% agar in IMDM with 10% FBS and overlaid onto 0.5% basal agar coat in 35 mm plates. The plates were incubated at 37 °C in a humidified atmosphere containing 5% CO_2_ for 21 days and colonies formed were visualized under an inverted microscope. Images were captured using Axiovert 200 inverted microscope (Carl Zeiss).

### *In vivo* tumourigenicity and PET

For animal study approved by Institutional Animal Ethics Committee (Project no. 05/2012), 1 × 10^7^ cells were injected subcutaneously in nude (ITOC-01) and NOD-SCID mice (ITOC-02, ITOC-03, ITOC-04). All experiments were performed in accordance with relevant guidelines and regulations of Institutional Animal Ethics Committee. The mice were housed in sterile pathogen-free environment and observed daily for appearance of tumors. FDG PET studies were performed for the xenografts established from these cell lines in nude and NOD-SCID mice (Supplementary Materials and Methods). Tumors from sacrificed mice were stained with hematoxylin and eosin (H&E) and images captured using Axio Imager.Z1 upright microscope (Carl Zeiss).

### Whole exome sequencing

Genomic DNA libraries were prepared using Nextera® Rapid Capture Enrichment Kit (Illumina) as per the manufacturer’s instructions and paired-end reads were generated using Illumina HiSeq 1500 platform. Approximately 37 Mb region of human genome comprising of 214,405 exons was captured. Detailed analysis provided in Supplementary Materials and Methods.

### TP53 mutational analysis

Genomic DNA was isolated from cells (Qiagen, 51104), fresh frozen tissue (PreAnalytiX, 767134) and formalin-fixed paraffin-embedded (FFPE) tissue (Qiagen, 56404) as per the manufacturer’s instructions. Primer sequences used for amplification of Exon 1–11 of TP53 gene were as described earlier^47^. Mutational analysis was carried out by bidirectional Sanger sequencing of PCR products on ABI 3730 capillary sequencer (Applied Biosystems).

### RNA sequencing

Total RNA was isolated from the 4 tumor cell lines and 3 normal buccal mucosa tissues using miRNeasy mini kit (Qiagen). RNA libraries were prepared using TruSeq RNA sample preparation kit (Illumina) as per the manufacturer’s instructions and paired-end reads were generated using Illumina HiSeq 2500 platform. Please refer to Supplementary Materials & Methods for detailed RNA-seq analysis. RNA-seq data was validated by qPCR (Supplementary Materials & Methods). Primer sequences are specified in Supplementary Table S8.

## Supplementary information

Supplementary Information

Dataset 1

Dataset 2

Dataset 3

## Data Availability

Data is available on reasonable request according to data protection policy in the ethics agreement.
