# Supplementary material for: Establishment and genomic characterization of gingivobuccal carcinoma cell lines with smokeless tobacco associated genetic alterations and oncogenic PIK3CA mutation
Source: Sci Rep. 2019 Jun 4;9:8272. doi: 10.1038/s41598-019-44143-0 (PMC6547758; doi:10.1038/s41598-019-44143-0)
Supplement: Supplementary file 1 — Supplementary Information [file 41598_2019_44143_MOESM1_ESM.pdf]

Supplementary Information

Establishment and genomic characterization of gingivobuccal carcinoma cell lines with smokeless tobacco associated genetic alterations and oncogenic *PIK3CA* mutation

Kshama Pansare[a], Nilesh Gardi[b], Sayee Kamat[a], Prerana Dange[a], Rahul Previn[a], Poonam Gera[a,c], Pradnya Kowtal[a,d], Kishore Amin[a,c], Rajiv Sarin[a,d]

[a]ICGC Lab, [b]Translational Research Lab, [c]Biorepository, [d]Sarin Lab, Advanced Centre for Treatment, Research and Education in Cancer, Tata Memorial Centre, Kharghar, Navi Mumbai – 410210, India

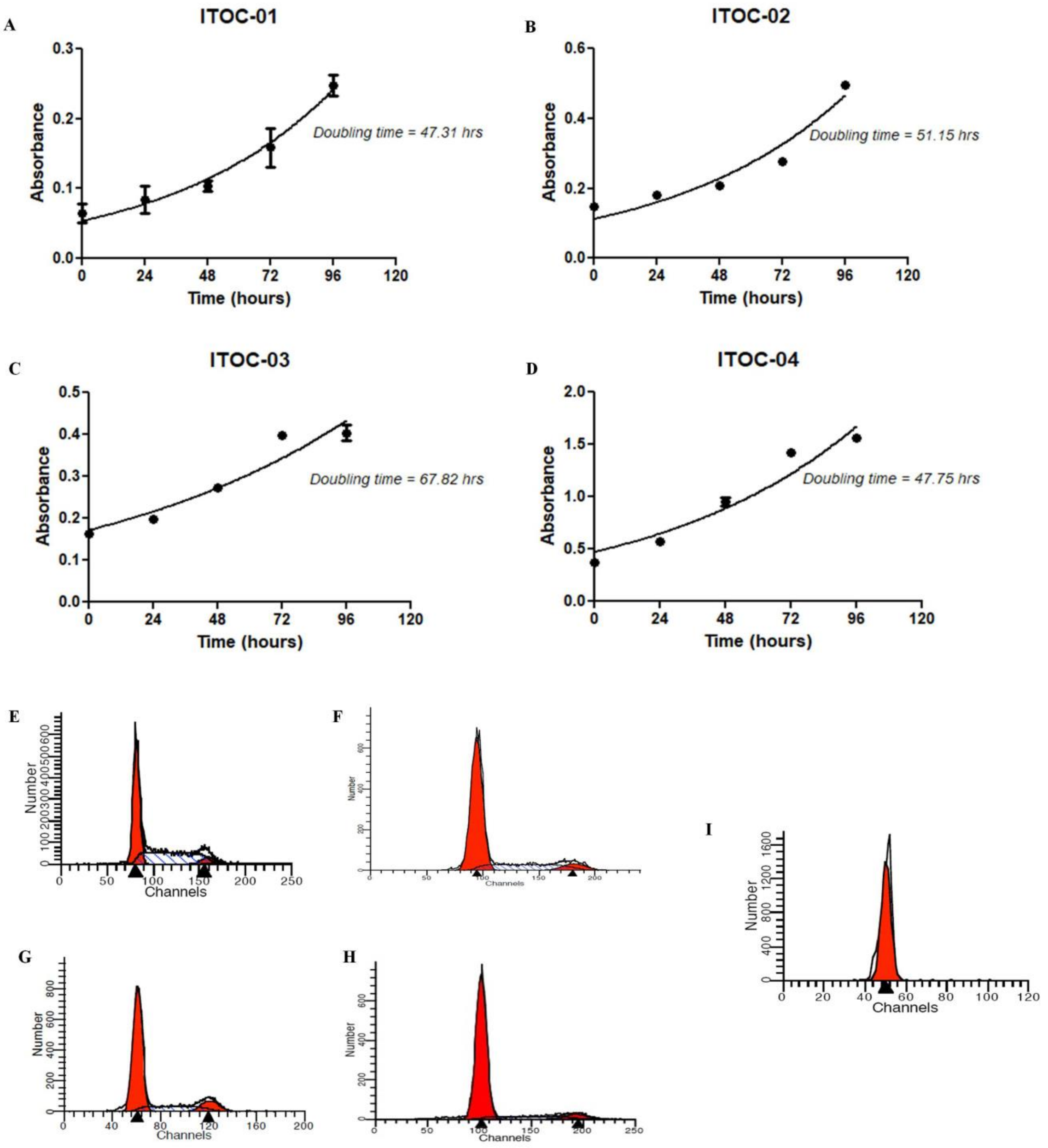

Supplementary Figure S1: Growth curve , doubling time and DNA histogram of OSCC tumor derived cell lines
A-D. Growth curve and doubling time of ITOC-01, -02, -03 and -04 cell lines were 47.31, 51.15, 67.82, 47.75 hours. Error bars represent the mean ± SEM (n=3). E-H. DNA histogram of OSCC cell lines. I. DNA histogram of lymphocyte control.

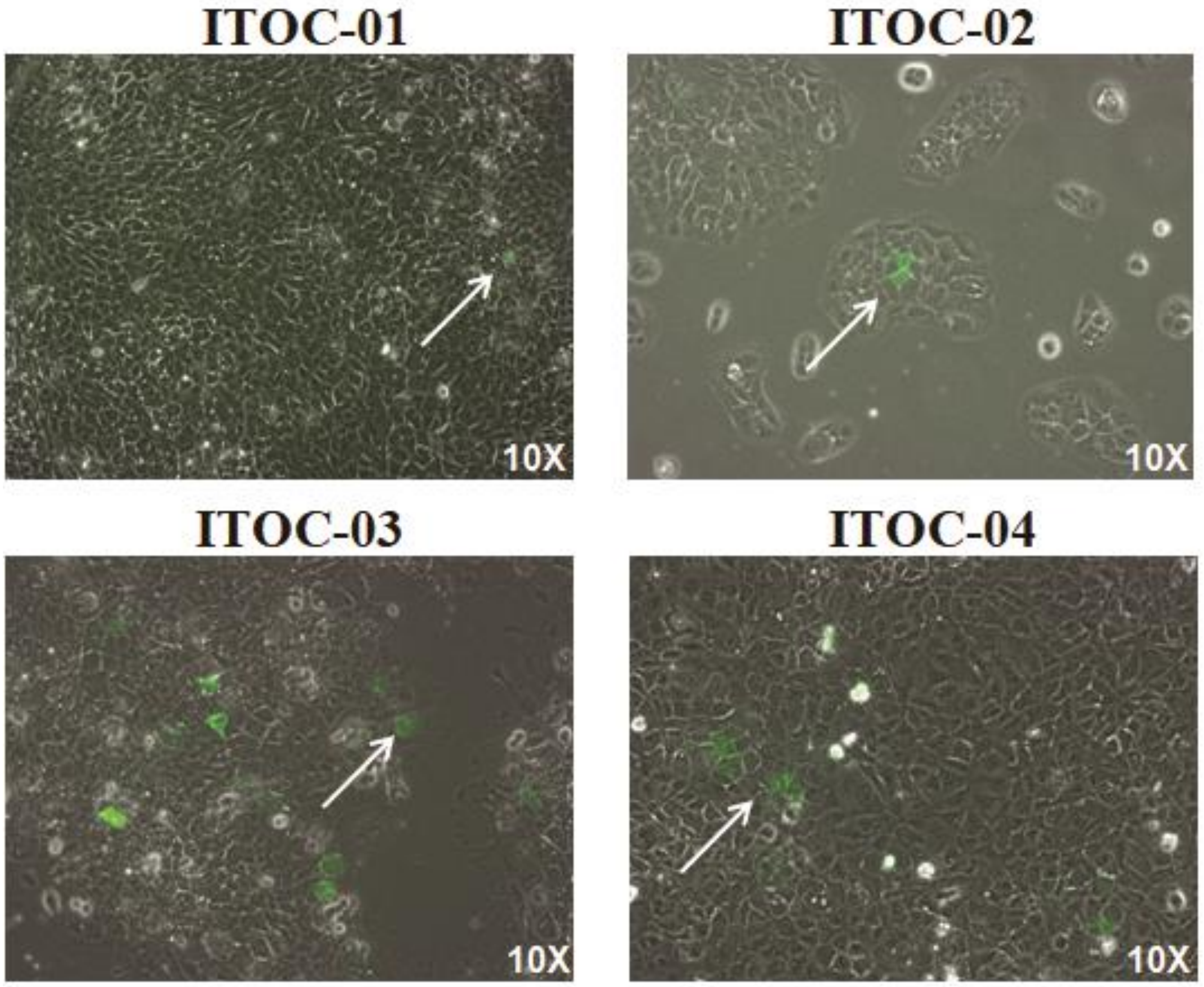

Supplementary Figure S2: Transfection efficiency assay of OSCC cell lines
Photomicrographs of cells captured 48 hours post-transfection by fluorescence microscope at 10X magnification. Representative transfected cells are indicated with white arrow. Transfection efficiency was observed to be low in ITOC-01 (1%) and ITOC-02 (6%) while high in ITOC-03 (30%) and ITOC-04 cell lines (20%).

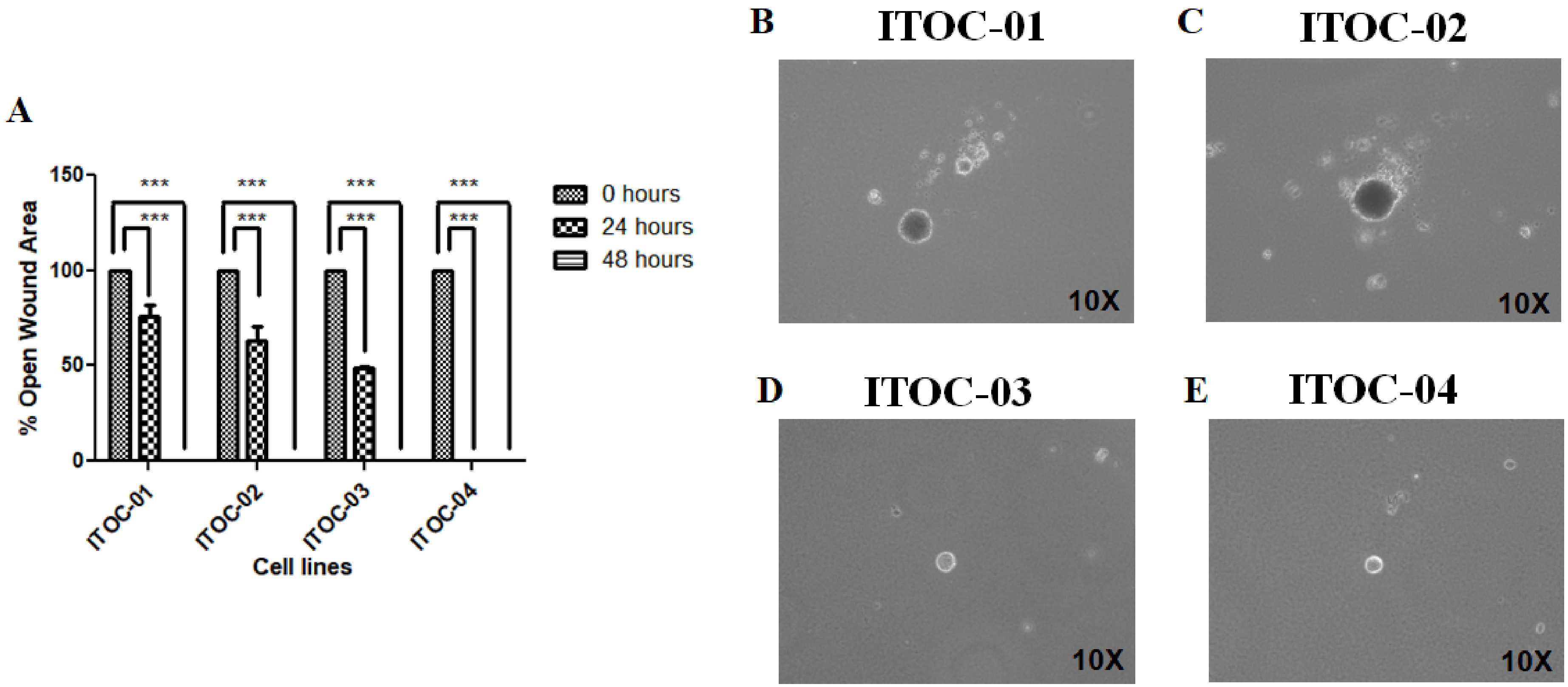

Supplementary Figure S3: Scratch assay and anchorage independent growth. A. Migration rate of OSCC cell lines at every 24 hours after inflicting scratch in a confluent cell monolayer. One-way ANOVA (analysis of variance) and Bonferroni post-tests results on control vs. time points. NB: *** - $P<0.001$. B-E. Soft agar colonies at Day 21.

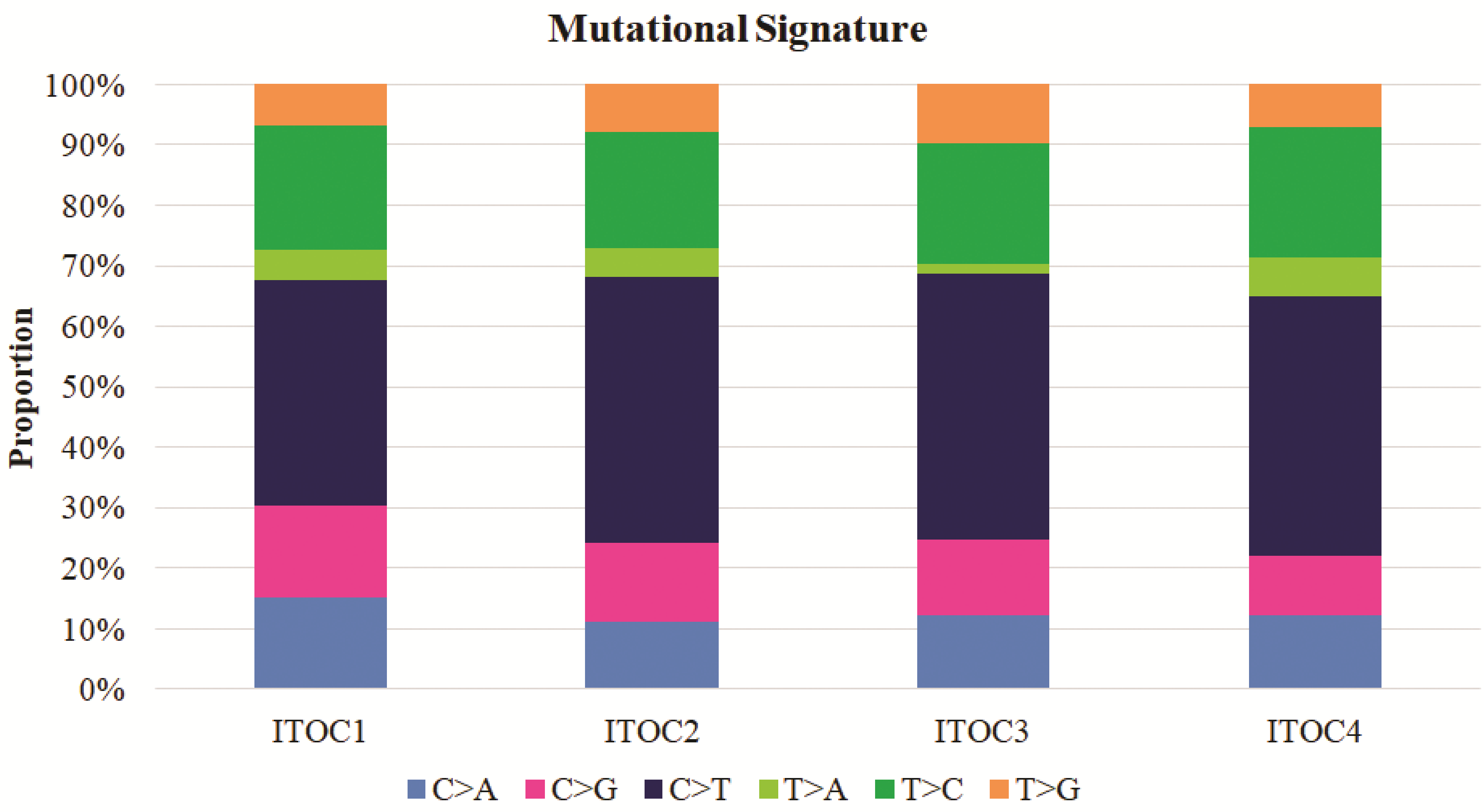

Supplementary Figure S4: Mutational signatures of OSCC cell lines
Bar graph representing proportion of transitions and transversions across all 4 OSCC cell lines. Higher proportion of C>T transitions followed by C>A and C>G transversions was seen.

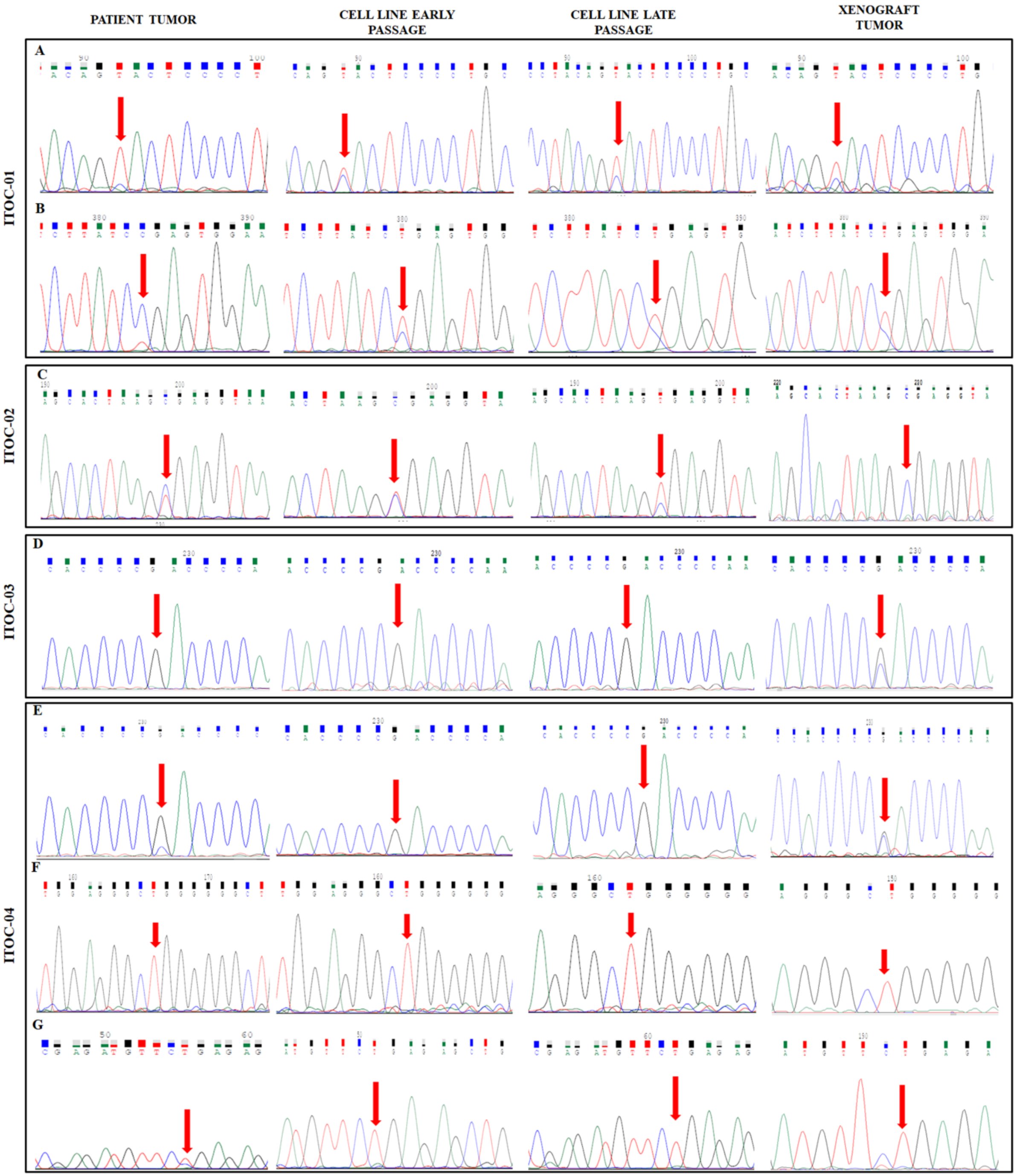

Supplementary Figure S5: TP53 mutational analysis

Mutational analysis of TP53 was performed on genomic DNA extracted from parent tumor, cell line at early passage (ITOC-01: P-10, ITOC-02: P-8, ITOC-03: P- 12, ITOC-04: P-9), cell line at late passage (ITOC-01: P-51, ITOC-02: P-48, ITOC-03: P-58, ITOC-04: P-57) and xenograft tumors. ITOC-01 cell line has 2 mutations, (A) TP53 exon 5+6, g.12655C>T; CGA>TGA; R196X nonsense mutation (B) TP53 exon 5+6, g.12364T>C; TAC>CAC; Y126H missense mutation, (C) ITOC-02 cell line has nonsense mutation at exon 8+9, g.13896C→T; CGA>TGA; p.R306X, (D,E) ITOC-03 and ITOC-04 cell line have mutations in intronic region of exon 2: IVS2+38C>G and ITOC-04 cell line also has 2 other mutations, (F) intronic region of exon 3: IVS3+36_52del16 (c.96+41_96+56del16) and (G) exon 10. g.16915C>T; CGA>TGA; p.R342X.

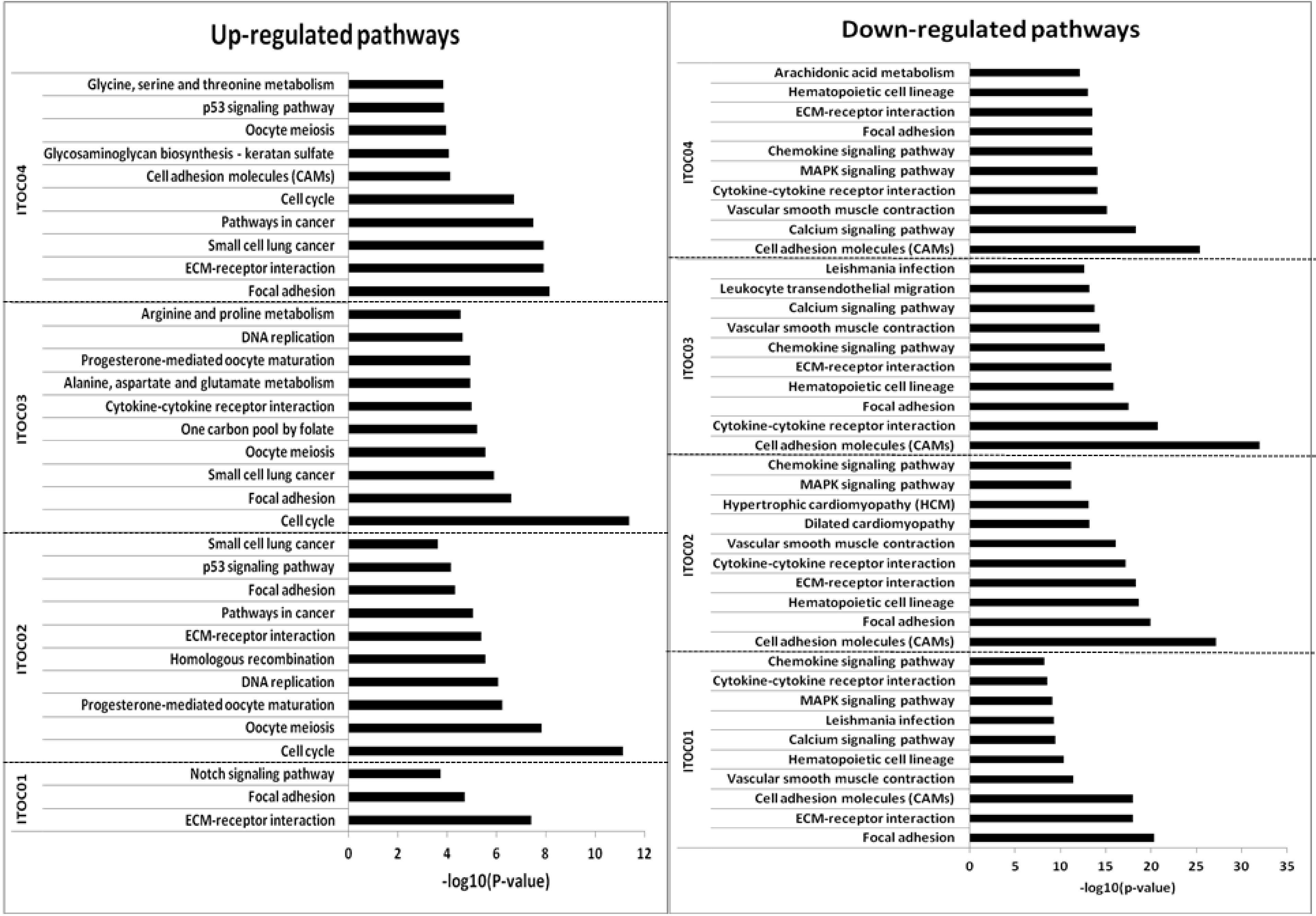

Supplementary Figure S6: Deregulated pathways in individual OSCC cell line

Pathway analysis of all individual OSCC cell lines vs. 3 normal tissue datasets identified commonly deregulated pathways in OSCC

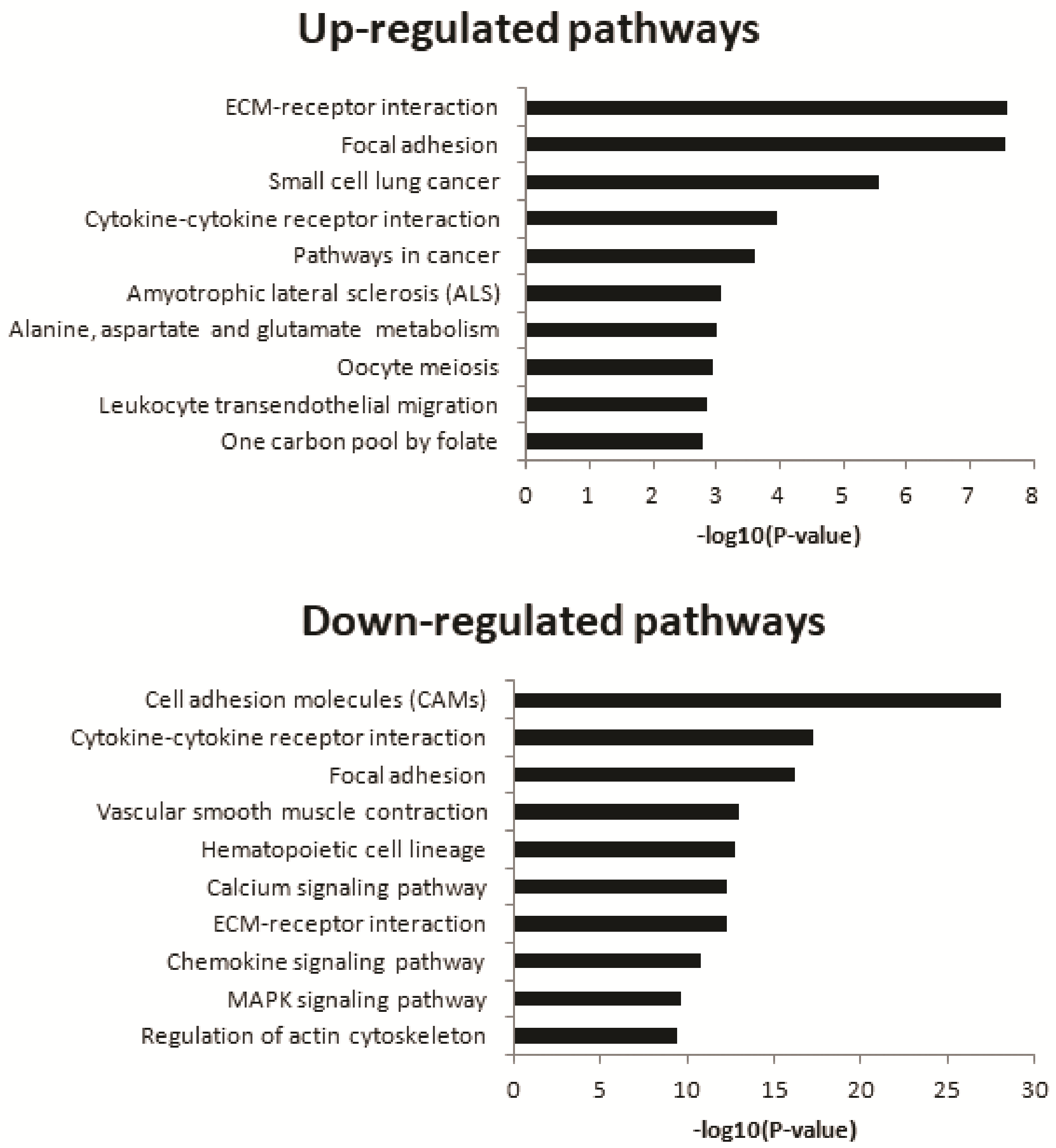

Supplementary Figure S7: Common pathways deregulated in OSCC-GB cell lines
KEGG GSEA pathway analysis of OSCC-GB cell lines; ITOC-01, ITOC-03 and ITOC-04 vs. 3 normal buccal mucosa tissue datasets.

Supplementary Table S1: Histomorphological analysis of primary and xenografted tumor tissue sections

hpf: high-power field

H&E stained tumor tissue sections were subjected to histological analysis. Comparison of primary and xenograft tumors was carred out using defined histomorphological parameters.

	**ITOC-01**		**ITOC-02**		**ITOC-03**		**ITOC-04**	
**Morphologic parameter**	**Patient tumor**	**Xenograft tumor**	**Patient tumor**	**Xenograft tumor**	**Patient tumor**	**Xenograft tumor**	**Patient tumor**	**Xenograft tumor**
Degree of cellular differentiation	Moderate to poorly differentiated	Moderate to poorly differentiated	Poorly differentiated	Moderate to poorly differentiated	Moderately differentiated	Moderate to poorly differentiated	Poorly differentiated	Poorly differentiated
Keratinization	20 - 50% cells keratinized	>50% cells keratinized	>50% cells keratinized	>50% cells keratinized	>50% cells keratinized	5 - 20% cells keratinized	<20%	20 - 50% cells keratinized
Pattern of keratinization	Individual cell keratinization and keratin pearls	Individual cell keratinization and keratin pearls	Individual cell keratinization and keratin pearls	Individual cell keratinization and keratin pearls	Individual cell keratinization	Individual cell keratinization	Individual cell keratinization	Individual cell keratinization and keratin pearls
Nuclear pleomorphism	Moderate to abundant nuclear pleomorphism	Moderate nuclear pleomorphism	Moderate and focally abundant nuclear pleomorphism	Minimal nuclear pleomorphism and focal abundant nuclear pleomorphism	Moderate nuclear pleomorphism	Abundant nuclear pleomorphism	Abundant nuclear pleomorphism	Moderate and focally abundant nuclear pleomorphism
Number of mitosis/hpf	0-2	0-2	0-1	0-2 (Focally 2-5)	0-2	0-2	1-3	0-1
Pattern of invasion	Pushing well delineated infiltrating borders and Small groups or cords of infiltrating cells	Small groups or cords of infiltrating cells	Pushing well delineated infiltrating borders and Small groups or cords of infiltrating cells	Small groups or cords of infiltrating cells	Small groups or cords of infiltrating cells	Pushing well delineated infiltrating borders and Small groups or cords of infiltrating cells	Marked and widespread cellular dissemination in small groups and/or in single cells	Pushing well delineated infiltrating borders
Lymphoplasmacytic infiltration	Moderate	Slight	None	Slight	Moderate	None	Slight	None
Grade of tumor	Poorly differentiated	Poorly differentiated	Poorly differentiated	Moderate to poorly differentiated	Moderately differentiated	Moderate to poorly differentiated	Poorly differentiated	Poorly differentiated

Supplementary Table S2: Genotypic characterization of four cell lines with STR markers

STR marker analysis at 9 different loci showing unique genotype of ITOC cell lines.

STR markers	Number of allele repeats			
	**ITOC-01**	**ITOC-02**	**ITOC-03**	**ITOC-04**
D5S818	10	9,10.1	10.1	10.1,12.3
D7S820	9.3, 12	8	8	9.3,12
D13S317	10, 12	13,13.3	11,14.3	11,13
D16S539	13	12.1,14	11,12.1	12.1
CSF 1PO	10, 12.1	10.3, 12	10.3,11.3	12,12
THO1	5	5.3,8.3	5.3,7.3	7,9.1
TPOX	7.3	9,10.3	10,10.3	10.3
vWA	15.2,16.1	15.2,19	15.2,17	13(15”), 17
AME (bps)	106.5,112.2	108	108,113	108.28

Supplementary Table S3: Classification of variants identified across OSCC cell lines

Types of variants	Cell lines			
	**ITOC-01**	**ITOC-02**	**ITOC-03**	**ITOC-04**
Total Variants	133454	105751	101272	98944
Exonic Variants	24150	21921	22576	22018
Nonsynonymous Variants + Indels	12365	11056	11455	11201
Putative Somatic Variants	6624	5810	6060	5865
ExAC South Asian allele frequency ≤ 0.1	1762	1274	1519	1365
Missense predicted deleterious by ≥ 5 tools	298	210	278	251
Stopgain	27	21	22	27
Splice site	20	15	14	25
Frameshift	24	18	28	9
Stoploss	0	1	1	0
Total deleterious mutation	369	265	343	312
Key genes mutated	TP53, NOTCH1, PIK3CA, SYNE2, PCLO	TP53, PYROXD2, PCMTD1, PABCP1	TP53, NOTCH1	TP53, NOTCH1, PCLO, FAT1, FAT3

Supplementary Table S6: Differentially expressed genes in OSCC cell lines

**Cell lines and tumor tissues**	**Number of up-regulated genes**	**Number of down-regulated genes**
ITOC01 *vs.* BM normal tissue	661	1627
ITOC02 *vs.* BM normal tissue	811	1997
ITOC03 *vs.* BM normal tissue	885	2343
ITOC04 *vs.* BM normal tissue	907	1950
OSCC cell line replicative *vs.* BM normal tissue	902	1492
BM cell line replicative *vs.* BM normal tissue	883	1686

Supplementary Table S8: Primer sequences for qPCR validation of RNA-Seq data

	Forward Primer	Reverse Primer
**KRT8**	CTCGAAGCAACATGGACAAC	CCAGCTCTACCTTGTTCATG
**VIM**	GTCAGCAATATGAAAGTGTGGC	GGTAGTTAGCAGCTTCAACGG
**MMP9**	CGCAGACATCGTCATCCAGT	AACCGAGTTGGAACCACGAC
**JAG1**	GCGTCATTGTGTTACCTGCGG	TCTTTGAGACGCTCCCCCTC
**IL6**	GTGGCTGCAGGACATGACAA	TGAGGTGCCCATGCTACATTT
**IL10**	TGAGAACAGCTGCACCCACT	GGCAACCCAGGTAACCCTTA
**PLA2G3**	ACACCAGGTTTCAGCAATGC	TCTCCAGCACGTTGAAGAAGG
**PLA2G4E**	TCCGCAAATTCCAGGAGGAG	ACGCTGATCTGACAGTTTGC
**PLA2G4F**	ATGGTGCTGTGAAGAACGTC	AGGTGTGTTTGTGAGGTTGG
**PTGIS**	AGAATTCAACCTGCGACGTG	TCAGGGTTCAGGAATCGGTTG
**TBXAS1**	AGCAGGTGTTGGTTGAGAAC	AGGACTGAAAGCAGACATCAGG
**GPX7**	TGACAGCAACAAGGAGAT	GTCTTCTCGCTTCAGTAGGA
**CYP2U1**	TACATAGAGACCCAGCCA	GCATTAGGCTCACAAACAT
**FOXP3**	CACCTGGCTGGCTGGGAAAATGG	GGAGCCCTTGTCGGATGAT
**KRT18**	TGAGACGTACAGTCCAGTCCTT	GCTCCATCTGTAGGGCGTAG
**VEGFA**	ATCTGCATGGTGATGTTGGA	GGGCAGAATCATCACGAAGT
**TGFBI**	TGGAAACCCACAACGAAATC	GGGTTCAGGTACCGCTTCTC
**AURKA**	TTAGGAATCGTGCAGGGGGA	AGGGAGGTTAAGGCACACCT
**AURKB**	TGGACCTAAAGTTCCCCGCT	ACCCGAGTGAATGACAGGGA
**HDAC9**	ACTGAAGCAACCAGGCAGTC	TTCACAGCCCCAACTTGTCC
**BCL2**	CCCCTCGTCCAAGAATGCAA	TCCCGGTTATCGTACCCTGT
**FOS**	AGACGAGAAGTCTGCGTTGC	AAGTCCAGGGAGGTCACAGA
**JUN**	TGGGTGTCCTACCCAGTGTT	AGGTTGGGGGCTACTTCTCA
**SOX2**	CACAACTCGGAGATCAGCAA	CCGGGAAGCGTGTACTTATC
**LEF1**	CTCCTGCAGCGGAGCGGAGATTACAGAG	GGCTCTAGAAGTAGACGAAAGAGGGGTTGG
**MYCN**	ACACCCTGAGCGATTCAGAT	CCACGTCGATTTCTTCCTCTTCA
**CYP2C19**	ACAACAAGCACAACCCTGAG	TTCCTGGACTTTAGCTGTGACC
**CCL2**	GTCTCTGCCGCCCTTCTGTGC	AACAGCAGGTGACTGGGGCAT
**LAMC2**	GAGCCAGATGCGACCGATGT	ACAGCGTTCTCCAGTGACAG
**IL23A**	TGTGCCCCGTATCCAGTGT	CGGATCCTTTGCAAGCAGAA
**β-actin**	AAGAGAGGCATCCTCACCCT	TACATGGCTGGGGTGTTGAA

Supplementary Methods

Transmission Electron Microscopy (TEM): Cells reaching 80% confluency were fixed with 3% glutaraldehyde for 1 hour at 4^oC and osmicated with 1% osmium tetraoxide for 1 hour at 4^oC. After dehydration with graded series of ethanol (30%, 50%, 70%, 90%, 100%; 15 mins each) cells were embedded in Araldite 502 resin (Ted Pella Inc, CA) and polymerized at 60^oC for 48 hours. Ultra-thin sections of 60-70 nm thickness were cut on ultramicrotome (Leica EM UC7, Austria), mounted on copper grids, contrasted with uranyl acetate and lead citrate and examined under a TEM (JEM 1010, JEOL, Tokyo, Japan) at 120 kV.

Positron Emission Tomography (PET): Isoflurane™ anesthesia was administered and mice were intravenously injected with 3.7 MBq (100 μCi) of ^{18}F-FDG after overnight fasting. PET studies were carried out 60 minutes post injection on the microPET scanner FLEX Triumph™ LabPET4 (Gamma Medica Ideas, Inc, Northridge, CA). Based on count rates, the PET scan was acquired for 20 - 30 minutes. Image reconstruction was performed using Maximum Likelihood Expectation Maximization (MLEM) iterative algorithm and analyzed using PMOD software, version 3.2. The Standard Uptake Value (SUV) was calculated by using the formula: SUV = Activity in region of interest/(Injected Dose/Body Weight).

Whole exome analysis: The Passing Filter read 150bp paired end sequences were mapped to the human reference genome (hg19, GRCh37) using Burrows-Wheeler Alignment 0.7.16a. Reads were sorted, indexed and PCR duplicates removed by using Picard tools (v. 2.10.0). BAM QC was used to check the quality of the input data using Qualimap tool (v 2.2.1). SAMtools mpileup (v. 1.6-1) was used to locate non-reference positions in tumour and germline samples using duplicate removed sorted bam files as input. Default setting were used in mpileup program except *mapping quality* >1 and *Base-alignment quality* computation disabled setting. In the absence of normal counterpart, mpileup files were further processed using VarScan2 *mpileup2cns* (version 2.4.3). Parameters used were *--min-var-freq 0.02,--min-coverage 8, --min-reads2*. Variants from unknown chromosome contigs were excluded. Latest version of ANNOVAR software was used to annotate remaining variants for functional annotation. Non-coding (intronic, intergenic, ncRNA, UTR etc.), synonymous and non-frameshift variants were excluded to restrict further analysis to only nonsynonymous, stopgain, stoploss, indel and splice site variants. In absence of matched germline variants from the blood or normal tissue of the same patient, we used a stringent filtering strategy to classify variants as germline or somatic. Variants previously reported as somatic in ICGC and COSMIC database were considered as somatic variants. Variants previously reported as germline by the dbSNP, ExAC or esp6500si databases were considered as germline variants. Somatic variants occurring with ≥0.1 allele frequency in the south Asian population of the ExAC database were also excluded for further analysis as these maybe germline variants. Missense somatic variants were considered as deleterious if 5 or more of the 11 functional prediction tools of ANNOVAR predicted them to be deleterious. TP53 mutations were validated by Sanger sequencing using primer sequences as described earlier [1].

RNA-seq analysis: Transcript level quantification were carried out using *Salmon* software tool version v0.8.1 [2] on raw FASTQ RNA-seq data. Transcriptome index building were performed on transcriptome reference index downloaded from *UCSC* browser followed by quantification of

transcripts. Ultra-fast read mapping procedure, potential changes in gene length across samples, less memory and disk usage makes *Salmon* different from other available tools. Further downstream analysis of differential gene identification were carried out using DEseq2 R Bioconductor package [3], which utilizes negative binomial generalized linear models. Transcript abundance files from *Salmon* were imported and conversion to gene level information was carried out using *tximport* R Bioconductor package [4]. Low count genes were removed before running DESeq2 function and class comparison was carried between each cell line *vs.* 3 Gingivobuccal normal tissues using replicative strategy. Log fold change and p-value (*Wald test*) were calculated for each gene for each comparison. Genes were called as differentially expressed if *Wald test* p-value is less than 0.05 and +2< log fold change < -2 criteria.

Real-time RT-PCR (qPCR): Total RNA isolated from these cell lines and normal buccal mucosa tissue using miRNeasy mini kit (Qiagen) and mRNA was reverse transcribed into cDNA using RevertAid RT kit (Thermo Scientific). Power SYBR Green PCR Master Mix (ABI), 10pM of each primer and 10ng of cDNA was used in qPCR reaction. The cDNA was analyzed in Quantstudio 12K flex (ABI) using following amplification conditions: denaturation at 95°C for 10 minutes and 40 cycles of amplification at 95°C for 15 seconds, 60°C for 1 minute.

References:

1 Liu, Y., Bodmer, W. Analysis of P53 mutations and their expression in 56 colorectal cancer cell lines. *Proc Natl Acad Sci U S A*.**103**, 976-981 (2006).
2 Patro, R., Duggal, G., Love, M., Irizarry, R., Kingsford, C. Salmon provides fast and bias-aware quantification of transcript expression. *Nat Methods*. **14**, 417-419 (2017).
3 Love, M., Huber, W., Anders, S. Moderated estimation of fold change and dispersion for RNA-seq data with DESeq2. *Genome Biol*. **15**, 550 (2014).
4 Soneson, C., Love, M., Robinson, M. Differential analyses for RNA-seq: transcript-level estimates improve gene-level inferences. *F1000Res*. **4**, 1521 (2015).
